# Impact of urinary catheter on resistance patterns and clinical outcomes on complicated urinary tract infection

**DOI:** 10.1007/s00192-022-05320-4

**Published:** 2022-08-22

**Authors:** Carlos Ernesto Lombo Moreno, Oscar Mauricio Muñoz Velandia, Cindy Alejandra Bonilla Sánchez, Juan Sebastián Montealegre Diaz, Javier Ricardo Garzón Herazo

**Affiliations:** 1 School of Medicine, Pontificia Universidad Javeriana, Cra 7 # 40-62 piso 7, 110231 Bogotá, Colombia; 2 Internal Medicine Department, Hospital Universitario San Ignacio, Bogotá, Colombia; 3 Infectology Department, Hospital Universitario San Ignacio, Bogotá, Colombia

**Keywords:** Colombia, Epidemiology, Microbiology, Urinary tract infection

## Abstract

**Introduction and hypothesis:**

Complicated urinary tract infection (cUTI) is highly prevalent and costly for health systems. The impact of the indwelling urinary catheter on etiologic agents and clinical outcomes has been poorly studied in Latin America.

**Methods:**

Cross-sectional study including patients with cUTI, with positive urine culture, treated at Hospital Universitario San Ignacio, Bogotá (Colombia) between 2017 and 2020. Clinical and microbiologic characteristics, treatments and outcomes are explored, comparing those with and without indwelling urinary catheter.

**Results:**

Seven hundred thirty-five patients with non-catheter-associated cUTI (NC-cUTI) and 165 with catheter-associated cUTI (CAUTI) were included. CAUTI group had a higher proportion of recurrent UTI (18% vs 33.3%, *p* < 0.001), ICU requirement (2.7% vs 8.5%, *p* < 0.001), longer hospital stay (6 vs 10 days, *p* < 0.001) and > 30 days unplanned readmission rate (5.8% vs 10.3%, *p* < 0.001). In the same group, we found a higher frequency of *Pseudomonas spp* (2.6% vs 9.4%, *p* < 0.001), *Enterococcus* spp. (2.4% vs 3.3%, *p* = 0.016), *Serratia marcescens* (0.6% vs 3.3%, *p* < 0.001) and *Citrobacter freundii* (0.5% vs 5.7%, *p* < 0.001). It implied a higher number of patients treated with fourth-generation cephalosporins (1.4% vs 4.8%, *p* = 0.004), ertapenem (32.9% vs 41.8%, *p* = 0.027) and carbapenems associated with a second antibiotic (1.9% vs 8.5%, *p* < 0.001).

**Conclusions:**

Patients with CAUTI have a higher frequency of resistant germs, require greater use of resources and have worse clinical outcomes than patients who do not require such devices. Measures should be strengthened to minimize its use, in both the hospital and outpatient setting.

## Introduction

Complicated urinary tract infection (cUTI) corresponds to a heterogeneous entity characterized by urinary tract infection (UTI) manifestations and risk factors associated with urinary tract structural anomalies, presence of a catheter or devices in the urinary tract, and comorbidities such as diabetes, neoplasms, immune disorders or isolation of multiresistant germs [1]. The UTI represents 1.8% of US hospitalizations with costs per hospitalization near to 10,000 dollars [2, 3]. Additionally, cUTI is associated with a high rate of therapeutic failure (26.6%)^4^, hospital readmission around 9%^2^ and 30-day mortality of 8.7% [4]. cUTI is associated with elevated costs and high health services requirement; therefore, cUTI is a relevant entity for health systems.

In Colombia, there are descriptions of microbiologic isolations in community-acquired UTI [5, 6] and UTI associated with health care in users of vesical catheter [7, 8]. However, in both Colombia and Latin America, there is a lack of information on the clinical manifestations, complications and resistance patterns in patients with catheter-associated UTI (CAUTI) and its differences with non-catheter-associated cUTI (NC-cUTI).

The aim of this study is to describe the clinical and microbiologic characteristics, treatments and outcomes in patients with cUTI and to compare CAUTI vs NC-cUTI, in a reference university hospital in Colombia.

## Methods

### Study design and participants

A cross-sectional study was carried out including patients with diagnosis of cUTI treated at the Hospital Universitario San Ignacio (HUSI) in Bogotá, Colombia, between January 2017 and May 2020. The inclusion criteria were: patients > 18 years old, urinary tract infection discharge diagnosis (ICD-10 code N10, N12, N13.6, N15.1, N15.9, N30.0, N30.8, N30.9 or N39.0), reported cUTI in clinical history, hospitalization ≥ 48 h, presence of clinical symptoms (example: dysuria, urgency, frequent urination, flank pain, positive closed fist percussion test, suprapubic pain or fever) and positive urine culture with ≥ 10^5^ colony-forming units (CFU)/ml and no more than two microorganisms isolated. The urine collection method depended on the presence or absence of urinary catheter or external devices (see below). A clean-catch sample was obtained on patients without urinary catheter. For users of urinary catheters or external devices who required replacement of the catheter, a new urinary sample was obtained through the catheter. Patients with neurogenic bladder were included if there was no clinical suspicion of another infection site and accomplished other cUTI diagnosis criteria. cUTI diagnosis criteria were defined according to diagnostic criteria recommended by European Association of Urology [9]. Pregnant women, patients referred to another hospital and those who completed hospital care in a home care service were excluded. The institutional research ethics committee approved the study (FM-CIE-0174-22).

Patient information was obtained from institutional electronic medical records. Sociodemographic data are systematically collected during patient care. Clinical presentation, attention year, comorbidities, antibiotics administered, intensive care unit (ICU) stay, length of hospital stay, 30 days hospital readmission after discharge and 30-day mortality were collected using standardized formats.

CAUTI was defined as cUTI in nephrostomy, suprapubic cystostomy or indwelling catheter users (external devices). NC-cUTI was defined as cUTI not associated with external devices. Immunosuppression was defined as: HIV infection, transplant, active neoplasia or prednisolone use ≥ 10 mg/day. Culture isolates and phenotypic resistance patterns were obtained according to microbiology laboratory reports. Resistance phenotypes were defined as follows [10–12]:

- **Natural pattern:** *Escherichia coli, Shigella, Salmonella enterica, P. mirabilis and Klebsiella* spp. isolation sensitive to beta-lactams.
- **Penicillinase-producing pattern**: Enterobacteria isolates with aminopenicillin, carboxypenicillin and low or intermediate resistance to ureidopenicillins
- **Penicillinase-hyperproducing pattern:** *Citrobacter koseri* and *amalonaticus* isolation or enterobacteria aminopenicillin and carboxypenicillin resistant and low or intermediate ureidopenicillin sensibility. Variable resistance levels to first- and second-generation cephalosporin (except cephamycin) and amoxicillin-clavulanic acid and diminished sensibility may be present.
- **AMPc pattern:** *Citrobacter freundii, Enterobacter spp., Providencia spp., Morganella morganii, Serratia spp, Hafnia alvei, Proteus vulgaris, P. penneri* and *Pseudomonas* spp. isolation or laboratory-confirmed first-, second- and third-generation cephalosporin resistance.
- **Extended-spectrum beta lactamase (ESBL) pattern:** Cephalosporin resistance (except cephamycin) with amoxicillin-clavulanate (AC) and carbapenem sensitivity, confirmed by laboratory.
- **Carbapenemase production pattern:** Microbiologic isolation resistant (or diminished sensitivity) to carbapenems and positive confirmatory test (Hodge test, EDTA or boronic acid test) [13]. Although non-enzymatic resistance may be present, positive detection is defined according to carbapenems resistance (or diminished sensitivity) and negative confirmatory test.
- **Others:** Gram-negative germs with alternative resistance patterns to those mentioned above, gram-positive germs and candida.

### Statistical analysis

Qualitative sociodemographic characteristics were described using absolute and relative frequencies. Mean and standard deviation were reported for quantitative variables with normal distribution and median and interquartile range for those variables with non-normal distribution. Variable normality was evaluated using the Kolmogorov-Smirnov test at a significance level of 5% ([*p* < 0.05). For the analysis, cUTI was divided into two groups: patients with NC-cUTI and CAUTI patients. Both groups were compared using a chi-square test, *t*-test or Mann-Whitney U test according to variable type. Statistical analysis was performed using the statistical program STATA (Stata Statistical Software: Release 16. College Station, TX: StataCorp LLC). Resistance profiles of the frequently isolated germs were plotted with Excel (Microsoft 365: Version 2203, Redmond, WA, USA).

## Results

Table 1 summarizes clinical and demographic characteristics of 735 patients with NC-cUTI compared with 165 patients with CAUTI. High immunosuppression prevalence was evidenced in both groups (35 vs 32.1%, *p* = 0.487). In the NC-cUTI group we found a higher proportion of men compared with CAUTI patients (39.7% vs 34.5%, *p* < 0.001). However, CAUTI patients had a higher proportion of recurrent UTI (18% vs 33.3%, *p* < 0.001), antibiotic use in the last 3 months (27.2% vs 41.8%, *p* < 0.001), ICU stay requirement (2.7% vs 8.5%, *p* < 0.001), longer hospital stay (median 6 vs 10 days, *p* < 0.001) and unplanned readmission at 30 days (5.8% vs 10.3%, *p* < 0.001) compared to NC-cUTI patients. No differences were found in the mortality rate 30 days after admission (1.8% vs 1.2%, *p* = 0.614).

**Table 1:** Clinical and sociodemographic characteristics in complicated urinary tract infection

	Total	NC-cUTI	CAUTI	*p*†
Variable	*n* = 900	*n* = 735	*n* = 165	
Age, years, median (ICR)	68 (53–79)	68 (54–80)	65 (49–77)	0.089
Male sex, *n* (%)	400 (44.4)	292 (39.7)	57 (34.5)	< 0.001
Hospitalization year, *n* (%)				
2017	240 (26.7)	202 (27.5)	39 (23.6)	0.275
2018	246 (27.3)	206 (28.0)	40 (24.2)	
2019	327 (36.3)	262 (35.5)	65 (39.4)	
2020	87 (9.7)	66 (9.0)	21 (12.7)	
Clinical presentation as pyelonephritis, *n* (%)	662 (73.6)	526 (71.5)	136 (82.4)	0.004
Urinary tract anatomic anomalies, *n* (%)	519 (57.7)	364 (49.5)	155 (93.9)	< 0.001
Immunosuppression, *n* (%)	310 (34.4)	257 (35.0)	53 (32.1)	0.487
Sepsis, *n* (%)	281 (31.2)	220 (29.9)	61 (37.0)	0.078
Recurrent UTI, *n* (%)	187 (20.8)	132 (18.0)	55 (33.3)	< 0.001
Antibiotic use previous 3 months, *n* (%)	268 (29.8)	200 (27.2)	69 (41.8)	< 0.001
Bacteremia, *n* (%)	167 (18.6)	129 (17.6)	38 (23.0)	0.102
Comorbidities, *n* (%)				
DM	239 (26.6)	214 (29.1)	26 (15.8)	0.001
CKD	186 (20.7)	130 (17.7)	56 (33.9)	< 0.001
Previous renal transplant	38 (4.2)	32 (4.3)	6 (3.6)	0.315
Stroke	41 (4.6)	16 (7.6)	10 (6.1)	0.305
Heart failure	42 (4.7)	35 (4.8)	7 (4.2)	0.775
Charlson index, mediana (RIC)	4 (2–6)	4 (2–6)	4 (2–7)	0.034
Charlson index ≥ 6, *n* (%)	242 (32.4)	188 (25.6)	54 (32.7)	0.061
ICU hospitalization, *n* (%)	34 (3.8)	20 (2.7)	14 (8.5)	< 0.001
ICU stay, days, median (RIC)	4 (2–5)	4 (3–5)	4 (2–5)	0.823
Hospitalization length stay, days median (RIC)	6.1 (4–10.6)	6 (2.5–9.3)	10 (6–15)	< 0.001
30-Day hospital readmission, *n* (%)	60 (6.7)	43 (5.8)	17 (10.3)	0.038
30-Day mortality rate, *n* (%)	15 (1.7)	13 (1.8)	2 (1.2)	0.614

*NC-cUTI*, non-catheter associated urinary tract infection; *CAUTI*, catheter-associated urinary tract infection; *ICR*, intercuartil range; *DM*, diabetes mellitus; *CKD*, chronic kidney disease; *UTI*: urinary tract infection; *ICU*: intensive care unit. †*p* comparing NC-UTI vs CAUTI

Table 2 summarizes microbiologic isolates, resistance patterns and treatment administered to evaluated patients. In the CAUTI group a second isolated germ was more frequent (6.7% vs 28.5%, *p* < 0.001) as was the isolation of *Pseudomonas* spp. (2.6% vs 9.4%, *p* <0.001), *Enterococcus* spp. (2.4% vs 3.3%, *p* = 0.016), *Serratia marcescens* (0.6% vs 3.3%, *p* < 0.001) and *Citrobacter freundii* (0.5% vs 5, 7%, *p* < 0.001). In contrast, *E. coli* isolation was less frequent (71.6% vs 38.2%, *p* < 0.001).

**Table 2:** Microbiologic isolates, sensitivity, treatment and related outcomes in complicated urinary tract infections

	Total	NC-cUTI	CAUTI	*p*†
Variable	*n* = 900	*n* = 735	*n* = 165	
Total isolated germs, *n* (%) *	996	784	212	
Etiologic agent				
*E. coli*	642 (64.2)	561 (71.6)	81 (38.2)	< 0.001
*Klebsiella*	112 (11.2)	84 (10.7)	28 (13.2)	0.308
*Proteus*	70 (7)	43 (5.5)	27 (12.7)	< 0.001
*Pseudomonas*	40 (4)	20 (2.6)	20 (9.4)	< 0.001
*Enterobacter*	20 (2)	13 (1.7)	7 (4.2)	0.13
*Enterococo*	31 (3.1)	19 (2.4)	12 (3.3)	0.016
*Morganella*	15 (1.5)	7 (0.9)	8 (3.8)	0.008
*Estafilococo*	8 (0.8)	5 (0.6)	3 (1.4)	0.261
*Serratia*	12 (1.2)	5 (0.6)	7 (3.3)	0.002
*Citrobacter*	16 (1.6)	4 (0.5)	12 (5.7)	< 0.001
*Candida*	3 (0.3)	1 (0.1)	2 (0.9)	0.647
*Otros*	27 (2.7)	22 (2.8)	5 (2.4)	0.722
Second germ isolated, *n* (%) **	96 (10.7)	49 (6.7)	47 (28.5)	< 0.001
Antibiogram sensitivity profile, *n* (%) *	996	784	212	
Natural	293 (29.4)	250 (31.9)	43 (20.3)	0.001
Penicillinases production	313 (31.4)	263 (33.5)	50 (24.2)	0.006
ESBL	178 (17.9)	148 (18.9)	30 (14.5)	0.111
AMPc	119 (11.9)	65 (8.3)	54 (25.5)	< 0.001
Carbapenemases resistance	38 (3.8)	21 (2.7)	17 (8.0)	< 0.001
Others	55 (5.5)	37 (4.7)	18 (8.5)	0.033
Antibiotic administered, *n* (%)				
First-generation cephalosporin	215 (23,9)	187 (25.4)	28 (17)	0.021
Second-generation cephalosporin	206 (22,9)	181 (24.6)	25 (15.2)	0.009
Third-generation cephalosporin	9 (1)	8 (1.1)	1 (0.6)	0.574
Fourth-generation cephalosporin	18 (2)	10 (1.4)	8 (4.8)	0.004
Ampicillin/sulbactam	13 (1,4)	10 (1.4)	3 (3.1)	0.748
Piperacillin/tazobactam	32 (3,6)	26 (3.5)	6 (3.6)	0.951
Ertapenem	311 (34,6)	242 (32.9)	69 (41.8)	0.027
Meropenem	21 (2,3)	16 (2.2)	5 (3)	0.512
Carbapenems associated with another antibiotic	28 (3.1)	14 (1.9)	14 (8.5)	< 0.001
Quinolones	16 (1.8)	15 (2)	1 (0.6)	0.208
Others	31 (3.4)	26 (3.5)	5 (3)	0.866

Acronyms: *NC-cUTI*, non-catheter-associated urinary tract infection; *CAUTI*, catheter-associated urinary tract infection; *ESBL*, extended spectrum betalactamase. †*p* compares NC-cUTI vs CAUTI. *Calculated according to total isolated germs. **Calculated according to total germ isolates in the same urine culture

Additionally, phenotypic resistance patterns were different. CAUTI patients had a greater AMPc isolation pattern (8.3% vs 25.5%, *p* < 0.001) and carbapenemase-producing germs (2.7% vs 8.0%, *p* < 0.001) compared to NC-cUTI.

**Fig. 1:**
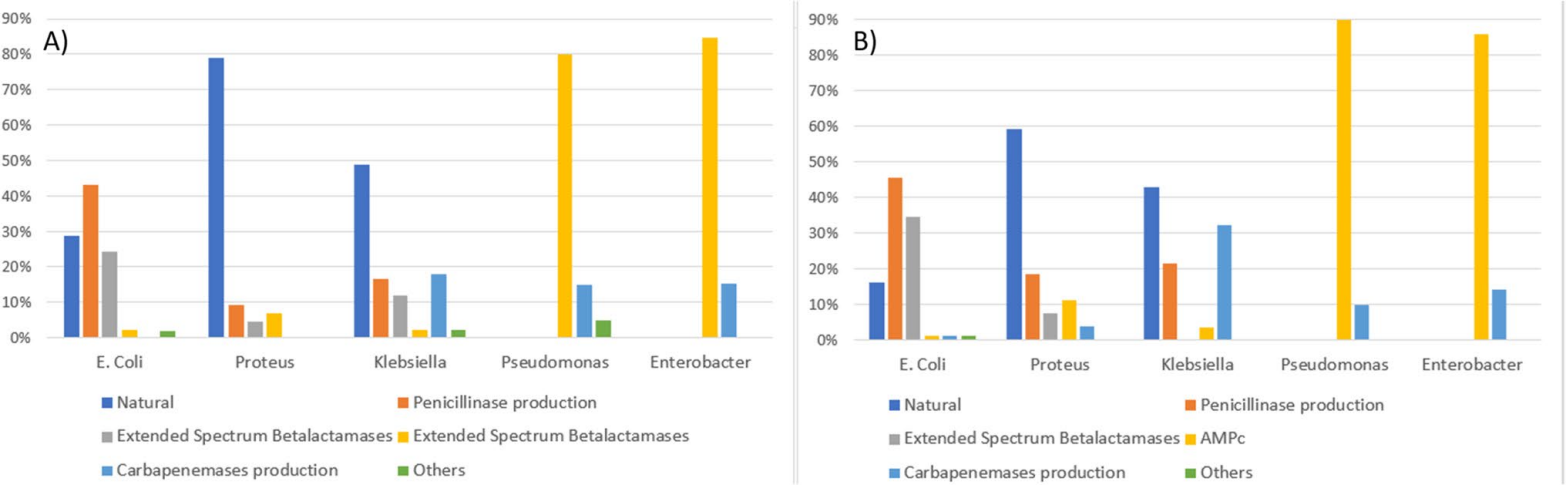
Complicated urinary tract resistance profile according to (A) non-catheter-associated urinary tract infection and (B) catheter-associated urinary Tract infection

Finally, targeted antibiotic treatment administration was different in both groups. NC-cUTI patients received first- (25.4% vs 17%, *p* = 0.021) and second-generation cephalosporins (24.6% vs 15.2%, *p* = 0.009) more frequently. Contrarily, CAUTI patients received fourth-generation cephalosporins (1.4% vs 4.8%, *p* = 0.004), ertapenem (32.9% vs 41.8%, *p* = 0.027) and carbapenems associated with a second antibiotic (1.9% vs 8.5%, *p* < 0.001) more frequently. Figure 1 shows the sensitivity profiles of the isolated germs for NC-cUTI and CAUTI.

## Discussion

In this study, we describe clinical and microbiologic characteristics, treatments and outcomes of patients with cUTI in a reference hospital in Bogota, Colombia. Our results show that patients with CAUTI presented: (1) a higher recurrent UTI rate, antibiotic use in the last 3 months and 30-day unplanned hospital readmission; (2) a higher percentage of AMPc and carbapenem resistance; (3) a higher requirement of fourth-generation cephalosporins, ertapenem and carbapenems associated with another antibiotic.

Demographic characteristics of our cUTI patients are similar to those reported worldwide, although some variations exist. Descriptive studies of cUTI show median ages between 65.1 and 73 years [3, 14, 15] and a similar male proportion [15, 16]. Other studies report sepsis or septic shock prevalence between 16% and 27% [15, 16], similar to our results (20.8%). Antibiotic use in the last 3 months was similar to European reports [4]. However, we found a lower ICU stay requirement (3.8%) compared to other reports in the USA (18.6 %) [16].

Regarding comorbidities, Charlson index is different between different populations. We found that 22.6% of our patients presented a Charlson index ≥ 6 [median 4 (IQR 2–6)], similar to a study developed in the USA that reported a Charlson index ≥ 5 in 18.22% [16]. Another study developed in the USA showed an average Charlson index of 1.08 [standard deviation (SD) 1.83] [2] while in Europe they reported an average Charlson index of 2.4 (SD 2.39) [15]. This difference could be explained because our institution is a reference hospital, treating more complex and comorbid patients.

On the other hand, hospitalization outcomes are similar. In this study, we found a length of stay of 6.1 [interquartile range (IQR) 4–10.6] days, similar to that reported in the USA (5, IQR 3–8) [15]. Thirty-day readmission rate (6.7%) was similar to European reports (4.53%) [15], and mortality was lower (1.7%) than that reported in the USA (2.78 %) [15] and Europe (5%) [15].

CAUTI patients had some clinical characteristics different from those reported in the international literature. In the USA, 66.39% of CAUTI patients were male [3], which is higher than the 34.5% reported in our results. In Colombia, a study carried out in two hospitals in Antioquia [8] showed 51.1% male patients. It is possible that the lower prevalence of males reported in our study is associated with HUSI condition as a cancer center reference hospital with higher requirement of catheters or external devices due to a malignant urinary tract obstruction.

Main cUTI isolation profiles are *E. coli* (64.2%), *Klebsiella* spp. (11.2%), *Proteus* spp. (7%) and *Enterococcus* spp. (3.1%). This profile is similar to international literature [1, 16, 17] and to that reported by the Bacterial Resistance Control Group in Bogotá (GREBO, in Spanish) in 2017 [18]. However, there are differences in the isolates found in patients with CAUTI compared to NC-cUTI. In the first group, we found a lower prevalence of *E. coli* and a higher number of AMPc constitutive germs (*Pseudomonas spp., Enterobacter spp., Serratia marcescens* and *Citrobacter freundii*). There was a similar result compared to the 25.8% of constitutive AMPc germs found in Europe [16] or the 22% of AMPc constitutive germ isolates found in a systematic review of patients managed in ICUs [19].

Sensitivity profile reported in patients with cUTI, regardless of the isolated germ, shows a high prevalence of ESBL germs (17.9%), without statistically significant differences between patients with CAUTI vs NC-cUTI (18.9% vs 14.5%, *p* = 0.111) (see Fig. 1). A study developed in 2010 with data from nine hospitals in Colombia reported a lower prevalence of ESBL laboratory confirmation, between 3.4 and 6.3% for *E. coli* and 3.4 to 17.2% for *K. pneumoniae* [20]. GREBO 2017 reports ceftriaxone resistance of *E. coli* and *Klebsiella* spp. with ESBL confirmation in 18.7% and 44.9%, respectively [18]. Our results present an intermediate resistance profile between these two studies of 26% in *E. coli* and 9% in *Klebsiella* spp. Our findings suggest there is a local increase in *E. coli* and *Klebsiella* spp. ESBL prevalence. Therefore, epidemiologic surveillance of these germs in cUTI should continue.

We found a carbapenems resistance (3.8%) higher than that reported in China (imipenem resistance of 0.5% for *E. coli* and 1.3% for *Klebsiella* spp.) [17]. A Colombian study in 2013 reported an *E. coli* resistance to ertapenem of 0% and *Klebsiella* of 6.9% [19]. GREBO 2017 [18] reported ertapenem resistance of *E. coli* of 1.5%, *Klebsiella* spp. of 23.3% and *Pseudomonas* spp. of 6.2%. Our results are similar to those found by the GREBO group (*E. coli* 0%, *Klebsiella* spp. 21%). Once again, these findings highlight the importance of epidemiologic surveillance.

Specifically, in CAUTI patients we found a high prevalence of carbapenem resistance (8.2%). With a higher prevalence in *Klebsiella* spp. (32%), although lower for *Pseudomonas* spp. (10%) compared with studies evaluating US ICUs (*Klebsiella* spp. 13% and *Pseudomonas* spp. 36%) [19]. Resistance profiles in CAUTI patients (AMPc and carbapenem resistance) explain the greater use of fourth-generation cephalosporins (4.8%), ertapenem (41.8%) and carbapenems associated with other antibiotics (8.5%). Therefore, CAUTI patients should receive a closer follow-up and periodic reassessment of catheter indication.

This is the first study in Latin America that reports the clinical characteristics, isolates and resistance profiles in cUTI according to the presence of a catheter or external devices. It increases the knowledge about the local microbiologic profile of our patients and provides important information for epidemiologic follow-up. Additionally, it is based on clinical diagnostic criteria for cUTI, thus facilitating clinical practice implementation of the results.

There are some limitations. A selection bias of patients with cUTI cannot be ruled out since the operational definition of this disease remains heterogeneous and could have been different at the moment of recording discharge diagnosis by the treating physician. However, we reviewed the recorded information and classified patients according to internationally accepted criteria. Also, this study was developed in a single center. Therefore, the external validity in other populations (especially in hospitals with less complexity) should be evaluated in the future. Lastly, we did not categorize the cUTI population according to whether it was community-based or healthcare-related, or by time since catheter or external device placement. This information was not reliably recorded in the medical records; therefore, this study is considered exploratory and will promote new studies to confirm our findings.

In conclusion, this study reports the clinical characteristics, isolates and resistance profiles in patients with cUTI. Patients with CAUTI were associated with a higher percentage of recurrent UTI, antibiotic use in the last 3 months, 30-day unplanned hospital readmission, higher prevalence of AMPc resistance profile, greater carbapenem administration, and greater fourth-generation cephalosporins and carbapenems associated with another antibiotic administration. Finally, empiric administration of ertapenem or fourth-generation cephalosporins in patients with CAUTI should be assessed in future studies.
